# Differential responses of fungal guilds to climatic effects mediated by changes in dominant vegetation

**DOI:** 10.1111/nph.71523

**Published:** 2026-08-24

**Authors:** Andrea Moravcová, Florian Barbi, Iñaki Odriozola, Tijana Martinović, Vendula Brabcová, Lenka Mészárošová, Ella Thoen, Antonín Machač, Petr Baldrian, Petr Kohout

**Affiliations:** ^1^ Institute of Microbiology of the Czech Academy of Sciences Videnska 1083 14200 Prague 4 Czechia; ^2^ Faculty of Science Charles University Viničná 5 128404 Prague 2 Czechia; ^3^ Department of Biosciences University of Oslo PO Box 1066, Blindern 0316 Oslo Norway

**Keywords:** climate change, fungal community assembly, fungal ecological guilds, plant–fungus interactions, species distribution modeling

## Abstract

- Climate is a major driver of fungal species distributions and community composition, yet disentangling the effects of climate‐mediated changes in dominant vegetation from other climatic effects remains a key challenge.
- To separate climatic effects mediated by dominant vegetation from other climatic effects on soil fungal communities, we analyzed soil fungal communities from eight European altitudinal gradients including sites with homogeneous and heterogeneous dominant vegetation. We combined community‐level analyses with species‐level hierarchical modeling to assess how different fungal ecological guilds respond to climate.
- Our results reveal clear contrasts among fungal ecological guilds. Ectomycorrhizal fungi showed significantly higher community turnover along the climatic gradient than any other group and responded only weakly to changes in dominant vegetation, whereas plant pathogens, root endophytes, and saprotrophic fungi showed consistently stronger responses under heterogeneous vegetation.
- The realized climatic niche of ectomycorrhizal fungi therefore appears only weakly constrained by dominant vegetation, whereas the distributions of plant pathogens, root endophytes, and saprotrophic fungi are more strongly associated with climate‐mediated vegetation change. Together, our findings demonstrate that fungal guilds differ markedly in the pathways through which climate shapes their distributions.

## Graphical abstract

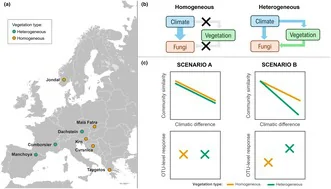

## Introduction

Climate change represents one of the most pressing global challenges of our time, with significant shifts in temperature and precipitation patterns expected to reshape ecosystems world‐wide. These changes will affect species distributions, interactions, and survival across all trophic levels, resulting in profound consequences for biodiversity and ecosystem functionality (Amundson *et al*., 2015; Baldrian *et al*., 2023). Among the many affected organisms, fungi play critical ecological roles in nutrient cycling, decomposition, and plant symbioses, making them essential to ecosystem functioning (Crowther *et al*., 2019). Therefore, climate‐driven shifts in fungal diversity and community composition are expected to have important consequences for ecosystem processes, including net primary production and soil carbon sequestration (Baldrian *et al*., 2022, 2023; Moravcová *et al*., 2023). However, predicting which fungal groups are most sensitive to climate change and how these responses will affect ecosystem functioning remains a major challenge.

Climate is widely recognized as a major driver of fungal species distributions and community composition at regional and continental scales (Větrovský *et al*., 2019; Baldrian *et al*., 2022). However, climatic variation is often accompanied by changes in vegetation and soil properties, making it challenging to disentangle their relative contributions to fungal community assembly (Větrovský *et al*., 2019). While research on various organisms, including insects (Kuczyk *et al*., 2021), vertebrates (Blaustein *et al*., 2010; Duclos *et al*., 2019; Kuczyk *et al*., 2021; Millington *et al*., 2022; Ghosh *et al*., 2024), and biotic interactions (Tylianakis *et al*., 2008), has demonstrated that climate‐driven changes often propagate through ecological interactions, the contribution of climate‐mediated vegetation turnover to fungal community assembly remains poorly understood. Vegetation plays a crucial role in shaping fungal habitats through its influence on organic matter composition, microclimatic conditions, and host associations (Classen *et al*., 2015; Urbanová *et al*., 2015).

The importance of vegetation turnover is likely to differ among fungal ecological guilds. Obligate biotrophic fungi, such as ectomycorrhizal (ECM) fungi, are tightly linked to plant communities and may be especially vulnerable to climate‐driven vegetation changes (Fernandez *et al*., 2017; Labouyrie *et al*., 2023). While ECM fungi are often characterized by relatively narrow climatic niches (Miyamoto *et al*., 2018; Větrovský *et al*., 2019), it remains unclear to what extent these patterns reflect direct climatic constraints vs. the climatic distributions of their host plants. Similarly, plant pathogens and root endophytes, which rely on specific host associations, are susceptible to climate‐induced shifts in plant community composition. By contrast, free‐living saprotrophic fungi, which drive decomposition processes, are generally less dependent on living host plants than biotrophic fungal guilds, although they often differ in their preferences for the type of litter and other organic substrates they decompose. Consequently, their responses may be influenced both by climatic conditions and by variation in substrate availability associated with vegetation and soil properties (Qin *et al*., 2023).

The objective of this study was to determine how climate‐mediated changes in dominant vegetation influence fungal community composition. Altitudinal gradients can serve as natural experiments for studying community and ecosystem responses to climate (Fukami & Wardle, 2005; McCain & Colwell, 2011). These gradients are characterized by predictable changes in temperature with increasing altitude, typically accompanied by shifts in precipitation and evapotranspiration (Körner, 2007). In temperate regions, these climatic gradients often drive a well‐documented pattern of vegetation zonation, where deciduous trees tend to dominate lower altitudes, mixed forests occur at mid‐altitudes, and coniferous species prevail in colder, higher‐altitude zones. These transitions are typically gradual rather than abrupt, reflecting continuous changes in temperature and other environmental variables along the gradient (Sundqvist *et al*., 2013). This vertical turnover of dominant tree species is primarily temperature‐driven, reflecting species‐specific thermal tolerances and physiological constraints (Körner, 2007; Sundqvist *et al*., 2013). However, not all mountain slopes exhibit this expected zonation; some can be dominated by a single tree species along the entire altitudinal gradient. Such a pattern can result from broad climatic tolerances of dominant species, absence of competing taxa, or site‐specific conditions such as soil properties, disturbance history, land use, or local microclimatic factors including moisture availability and radiation exposure (Körner, 2007; McCain & Colwell, 2011).

We conducted an extensive sampling campaign along eight altitudinal gradients in European mountain systems, including transects with relatively constant dominant vegetation along the entire gradient (homogeneous vegetation) and transects where dominant vegetation composition changed with elevation (heterogeneous vegetation). This design allowed us to compare fungal responses to climatic variation under contrasting levels of dominant vegetation turnover and to evaluate the extent to which climate‐mediated changes in dominant vegetation composition contribute to fungal community turnover. We hypothesized that fungal guilds differ in the extent to which their community composition is associated with climate‐mediated changes in dominant vegetation composition. Specifically, we expected plant‐associated fungi, including ECM fungi, plant pathogens, and root endophytes, to exhibit stronger compositional turnover along climatic gradients accompanied by changes in dominant vegetation composition (heterogeneous vegetation) than along gradients with relatively constant dominant vegetation (homogeneous vegetation). By contrast, we expected saprotrophic fungi to show more similar responses across both gradient types because they are less directly dependent on living host plants, although their distributions may still be influenced by vegetation‐associated changes in substrate availability and soil conditions. By comparing homogeneous and heterogeneous vegetation gradients, we therefore aimed to assess whether the importance of dominant vegetation turnover in shaping fungal community composition differs among fungal ecological guilds.

## Materials and Methods

### Study design

To evaluate the role of climate‐mediated changes in dominant vegetation composition in shaping fungal communities, we used natural variation in vegetation structure along altitudinal gradients. Because altitudinal gradients are associated with variation in multiple environmental factors, including geological substrate, soil development, and seasonality (Pickett, 1989), a single gradient would not allow robust inference about the relative importance of vegetation turnover. To address this limitation, we studied multiple spatially distant altitudinal gradients spanning *c*. 100–1750 m asl (Supporting Information Fig. S1) across several European mountain systems spanning diverse environmental conditions (Sundqvist *et al*., 2013).

**Fig. 1:**
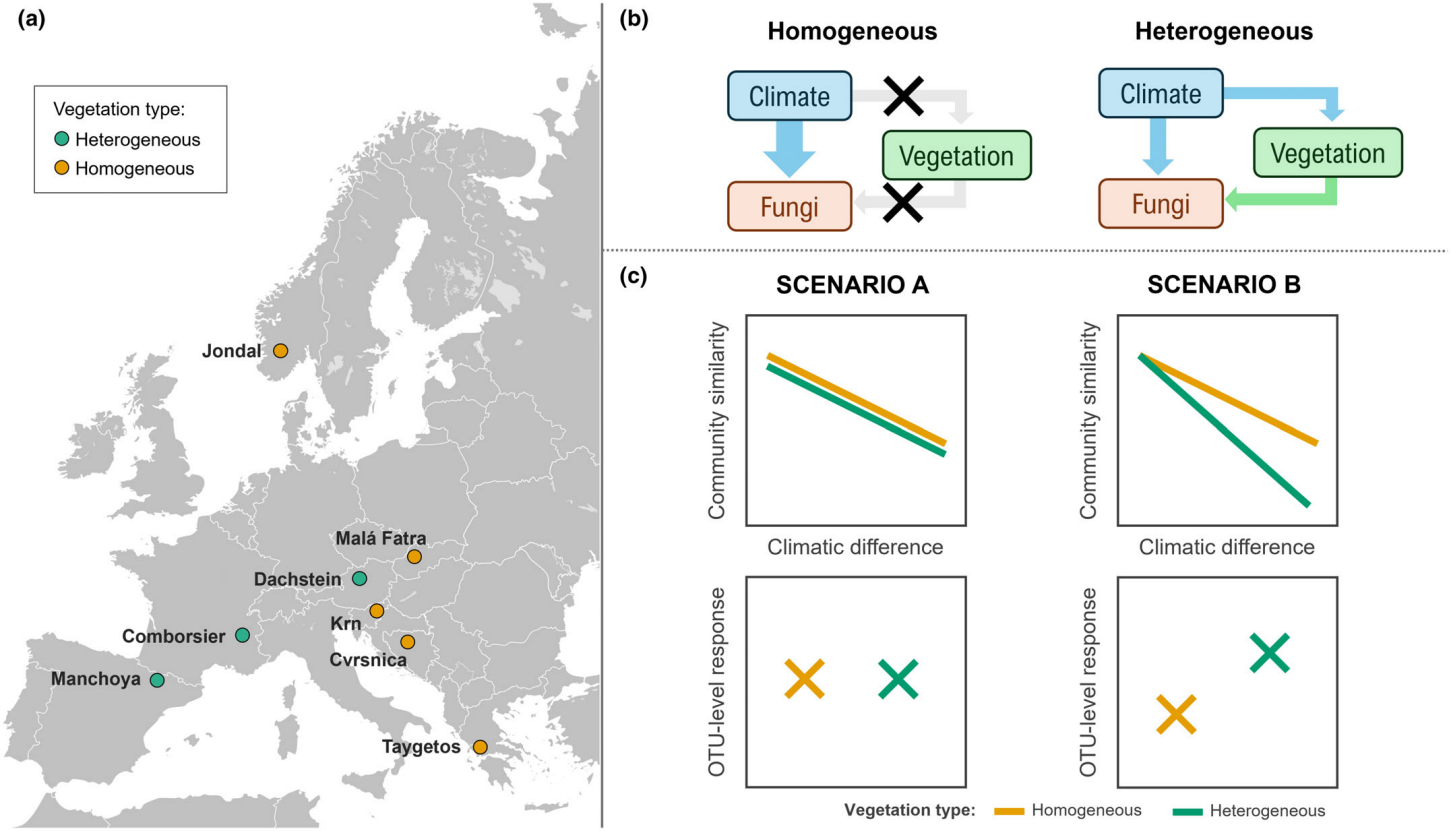
Sampling design and conceptual framework for assessing the contribution of changes in dominant vegetation to climatic effects on fungal comunities. (a) Sampling design and locations in Europe from the southernmost to the northernmost location: Taygetos (Greece), Manchoya (Spain), Cvrsnica (BIH), Comborsier (France), Krn (Slovenia), Dachstein (Austria), Malá Fatra (Slovakia), Jondal (Norway). Colors indicate vegetation type at the transect level: homogeneous vegetation (yellow) and heterogeneous vegetation (green). (b) Path diagrams illustrating the influence of the distinction between gradients with homogeneous vs heterogeneous vegetation on climatic effects on fungi. Under homogeneous vegetation, climatic effects mediated by changes in dominant vegetation are effectively closed; therefore, the effects mediated by other routes remain. By contrast, under heterogeneous vegetation, climatic effects also include the effects of changes in dominant vegetation. Note that the direct arrow from climate to fungi includes any direct or indirect effects with the exception of those mediated by dominant vegetation. (c) Schematic representation of two potential outcomes of fungal responses to climatic gradients. Scenario A represents similar fungal responses to climatic gradients under homogeneous and heterogeneous vegetation, indicating a limited contribution of dominant vegetation turnover to fungal community turnover. Scenario B represents stronger fungal responses under heterogeneous vegetation than under homogeneous vegetation, indicating that the climatic effects mediated by changes in dominant vegetation composition contribute substantially to fungal community turnover. The conceptual framework is illustrated at both the fungal community level (community similarity) and species level (absolute response of fungal operational taxonmic units (OTUs) to climatic distance).

Specifically, we distinguished between two types of transects based on the degree of dominant vegetation turnover along the gradient: (1) homogeneous vegetation transects, where the dominant tree species remained relatively constant across elevations, and (2) heterogeneous vegetation transects, where dominant vegetation composition changed with elevation following the expected altitudinal zonation pattern (Fig. 1a). In transects with heterogeneous vegetation, climate may influence fungi through multiple pathways, including effects mediated by the dominant vegetation. By contrast, in transects with homogeneous vegetation, these vegetation‐mediated pathways are effectively blocked, and climate can affect fungi only through other routes, including direct effects (Fig. 1b). Therefore, comparing these two types of gradients allowed us to disentangle climatic effects mediated by dominant vegetation from other climatic effects.

We considered two alternative expectations that may apply to fungal guilds with distinct ecological characteristics (Fig. 1c). Under the first scenario (Scenario A), fungal community turnover along climatic gradients is largely independent of dominant vegetation turnover. In this case, we would expect similar relationships between climatic variation and fungal community composition in homogeneous and heterogeneous vegetation gradients. Under the second scenario (Scenario B), climate‐mediated changes in dominant vegetation composition contribute substantially to fungal community turnover. In this case, we would expect stronger compositional changes along heterogeneous vegetation gradients than along homogeneous gradients experiencing comparable climatic variation.

### Sampling design

The locations, soil sampling design, and post‐sampling management were previously described in Barbi *et al*. (2025). Briefly, sampling was conducted at three, four, or five elevational sites along each gradient, with the uppermost site situated between 100 and 200 meters below the estimated tree line. At each elevational site, three sampling plots were established, resulting in a total of 99 plots across the study (Table S1). Plots within each elevation level were selected to represent the dominant local forest vegetation. At each plot, five soil cores (4 cm in diameter, 10 cm in depth) were collected using separate corers for each core, positioned centrally, and 2 m apart in the cardinal directions (Martinović *et al*., 2021). Soil cores were stored at 4°C and processed within 24 h. Litter, soil below a 10 cm depth, stones, and large roots were removed. The five cores per plot were then homogenized into a single composite sample, resulting in 99 composite samples across the study, freeze‐dried and stored at −20°C until further analyses.

### Plant survey

We performed a detailed survey of the tree cover composition within *c*. a 50 m radius on each sampling plot (Table S2). Transects that were categorized as having homogeneous dominant vegetation were dominated by a single tree species, either *Fagus sylvatica*, *Betula pubescens*, or *Pinus sylvestris*. Conversely, transects with heterogeneous dominant vegetation were typically dominated by *Fagus sylvatica* in lower elevational sites, which was replaced by *Picea abies* and *Pinus* sp. in higher elevational sites. Some transects with heterogeneous vegetation also included mixed vegetation at mid‐elevations. Transects with homogeneous vegetation included Jondal, Malá Fatra, Krn, Cvrsnica, and Taygetos. Transects with heterogeneous vegetation included Dachstein, Comborsier, and Manchoya (Fig. 1a).

### Soil chemistry analysis

Each soil sample was analyzed for pH, carbon (C) and nitrogen (N) contents, available phosphorus (P), and calcium (Ca). All analyses were performed by an external laboratory at the Institute of Botany of the Czech Academy of Sciences (Průhonice, Czech Republic), as previously described in Barbi *et al*. (2025). Briefly, soil pH was measured using a WTW Multilab 540 instrument in a 1 : 10 (w/v) suspension of soil and deionized water. C content was determined by sulphochromic oxidation, and total N was analyzed using a Carlo Erba EA 1108 elemental analyzer (Italy). Available P was quantified following Mehlich 3 extraction, with subsequent spectrophotometric measurement at 630 nm using a UV‐400 Unicam spectrophotometer (Thermo Scientific, Waltham, MA, USA). Ca content was analyzed in mineralized samples by atomic absorption spectrometry (ContrAA 700, Analytik Jena), with lanthanum chloride added to suppress phosphate and sulfate interference.

### Climatic data

**Fig. 2:**
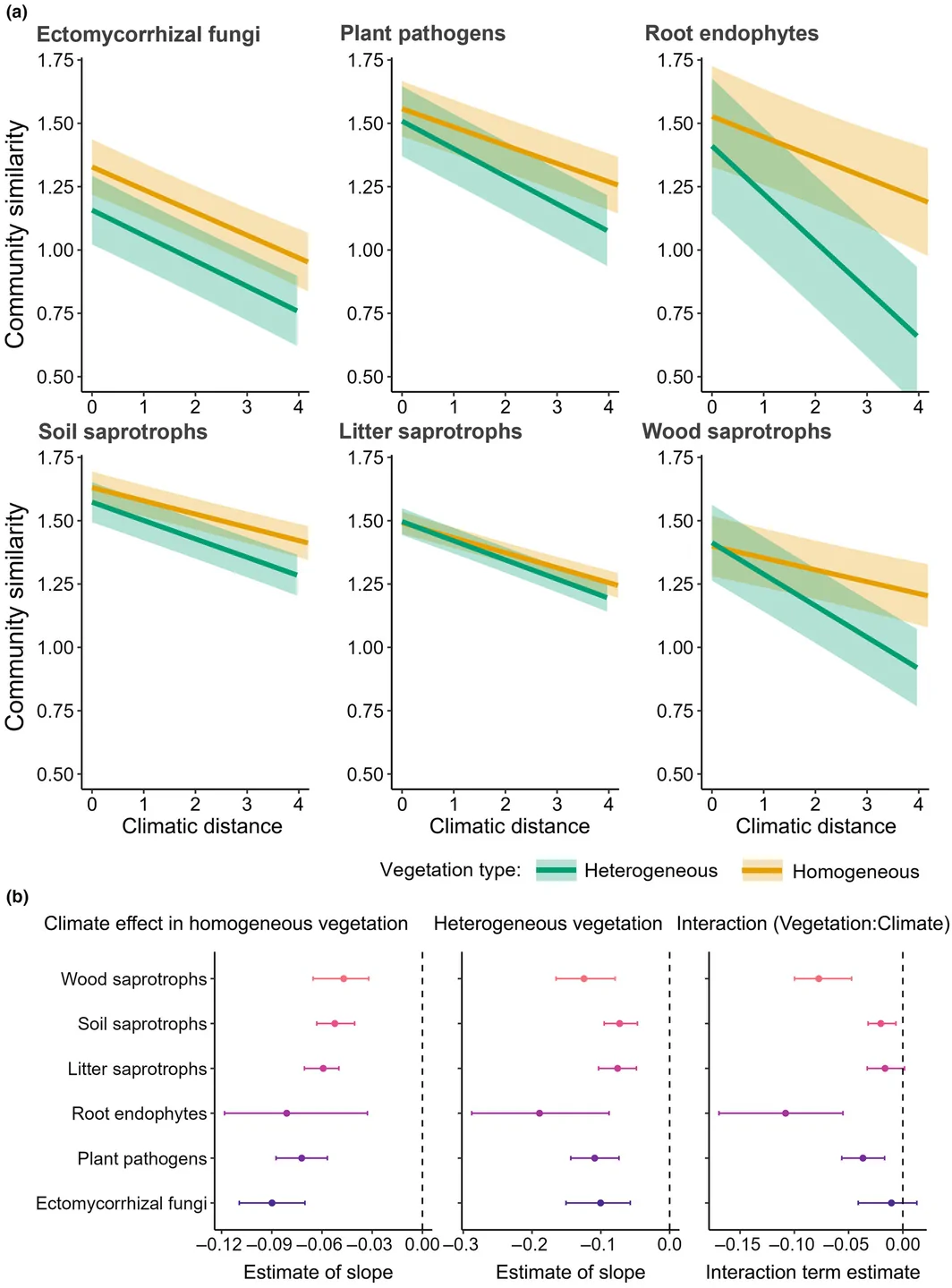
Fungal community responses to climatic distance across ecological guilds under homogeneous and heterogeneous vegetation. (a) Model‐predicted relationship between climatic distance and fungal community similarity along altitudinal gradients in heterogeneous (green) and homogeneous (yellow) vegetation. The y‐axis shows log_10_‐transformed community similarity values, calculated as log_10_((1 − Bray–Curtis dissimilarity) × 100 + 1). Shaded areas represent 95% bootstrap confidence intervals. Panels show results for different fungal ecological guilds. (b) Estimated strength of fungal community response to climate (slope estimates), categorized by ecological guilds. The three plots represent: (left) slope estimates for homogeneous vegetation, (middle) slope estimates for heterogeneous vegetation, and (right) the interaction effect between climatic distance and vegetation type. Confidence intervals indicate uncertainty in the estimates; non‐overlapping 95% confidence intervals are interpreted as significant differences in climate responses among fungal ecological guilds.

Climate data were obtained from the CHELSA database (Karger *et al*., 2017), specifically mean annual temperature (MAT) and mean annual precipitation (MAP), which represent the 30‐yr climate normal (1979–2013). While the CHELSA database generally provides more accurate climate estimates for mountainous regions compared to alternatives like WorldClim (Fick & Hijmans, 2017), its spatial resolution remains insufficient for the needs of our study. In several cases, we found that two elevational sites were assigned the same MAT value despite being separated by 200–300 m in elevation. To address this limitation, we applied a calibration procedure to refine the climatic variables along each elevational gradient. Specifically, we constructed a regression line between MAT and elevation for each gradient and used this relationship to recalibrate MAT values based on the elevation of individual sampling points. These adjustments ensured that the expected temperature lapse rate of *c*. 2°C per 200 m of elevation was maintained, improving the accuracy of the climate data used in our analyses. To assess the reliability of the values obtained from the calibrated CHELSA dataset, we conducted *in situ* measurements of air and soil temperature using TMS‐4 probes (TOMST, Czechia; Wild *et al*., 2019) at three studied locations, namely Dachstein, Comborsier, and Krn (Fig. 2). We calculated MAT in the air (2 and 15 cm above ground) and in the soil (2 cm below ground) from TMS‐4 probes using the ‘myclim’ package in R (Man *et al*., 2023). Besides that, we also included climatic data obtained from ForestClim (Haesen *et al*., 2023) in this comparison. We found tight correlations between the *in situ* air and soil temperature measurements and CHELSA rather than ForestClim data. The *in situ* measurements and CHELSA data showed a negative linear correlation along elevation with a similar function slope (Fig. S2).

### Molecular and bioinformatic analysis

The composition of fungal communities was assessed using metabarcoding techniques, following the protocol detailed in Barbi *et al*. (2025). Briefly, genomic DNA was extracted from 250 mg of soil per sample using a modified Miller method (Sagova‐Mareckova *et al*., 2008), and the fungal ITS2 region was amplified in triplicate via PCR using the universal fungal primers gITS7 (Ihrmark *et al*., 2012) and ITS4 (White *et al*., 1990), tagged with sample‐specific molecular identifiers (MIDs). Each PCR reaction contained 5 μl of 5× buffer and 5 μl of 5× Q5 High GC Enhancer (New England Biolabs, Inc., Ipswich, MA, USA), 0.5 μl of 10 mM PCR Nucleotide mix (Bioline, London, UK), 1.5 μl of 10 mg ml^−1^ BSA (GeneON), 0.25 μl of Q5 High‐Fidelity DNA polymerase (New England Biolabs, Inc.), 1 μl of each 10 μM forward and reverse primer (Sigma‐Aldrich), 9.75 μl of H_2_O, and 1 μl of template DNA, resulting in a total reaction volume of 25 μl. PCR conditions were as follows: an initial denaturation at 94°C for 5 min; 30 cycles of 30 s at 94°C, 30 s at 56°C, and 30 s at 72°C; followed by a final extension at 72°C for 7 min. Amplified DNA from triplicate reactions was pooled, purified, quantified, and prepared for sequencing using the TruSeq PCR‐free Kit (Illumina). Sequencing was performed on an in‐house Illumina MiSeq platform, generating paired‐end reads (2 × 250 bp).

Bioinformatic processing was conducted using the SEED 2.0 pipeline (Větrovský *et al*., 2018). Paired‐end reads were merged, quality‐filtered, and screened for chimeric sequences. The ITS2 region was extracted using ITSx v.1.0.8 (Nilsson *et al*., 2010), and sequences were clustered into operational taxonomic units (OTUs) at 97% similarity using the UPARSE algorithm (Edgar, 2013). Quality filtering was performed using an average Phred score threshold of 30, and chimeric sequences were removed before clustering. Taxonomic assignments were conducted via BLASTn against the UNITE database (v.10; Nilsson *et al*., 2019), and non‐fungal sequences were excluded. Functional guilds were assigned using the FungalTraits database (Põlme *et al*., 2020), following the classification criteria described in Barbi *et al*. (2025). Fungal OTUs were assigned to a putative functional group based on their genus‐level taxonomy and primary lifestyle. According to the FungalTraits database, OTUs were categorized into ectomycorrhizal fungi, root endophytes, plant pathogens, and saprotrophic guilds (soil, litter, and wood saprotrophs) based on their dominant ecological function. Full sequencing data are deposited in the Sequence Read Archive (SRA) under BioProject PRJNA869039.

### Statistical analysis

We assessed the extent to which climatic effects mediated by changes in dominant vegetation composition contribute to fungal responses along climatic gradients at both the community composition and OTU levels. To evaluate differences among fungal ecological guilds, all analyses were conducted separately for ECM fungi, plant pathogens, root endophytes, soil saprotrophs, litter saprotrophs, and wood saprotrophs.

#### Community composition analyses

To assess whether fungal community turnover along climatic gradients differs between homogeneous and heterogeneous vegetation, we first quantified community dissimilarity between sample pairs within each gradient using the Bray–Curtis dissimilarity index calculated from Hellinger‐transformed OTU matrices derived from raw sequence count data. The Hellinger transformation first converts raw counts to relative abundances and then applies a square‐root transformation, reducing the influence of highly abundant taxa while increasing the contribution of less abundant taxa to measures of community dissimilarity. To facilitate interpretation, Bray–Curtis dissimilarity values were converted to similarity using the transformation (1 − Bray–Curtis dissimilarity), thereby matching the classical distance‐decay framework based on community similarity. We then applied a log_10_ transformation to community similarity to linearize its relationship with climatic distance. This transformation corresponds to the exponential distance‐decay model, in which community similarity declines proportionally as environmental distance increases. Model assumptions of normal distribution and homoscedasticity of residuals were assessed visually. Climatic distance was calculated as the Euclidean distance between sample pairs based on calibrated mean annual temperature (MAT_calibrated) and MAP. Similarly, soil distance was calculated as the Euclidean distance between sample pairs based on standardized (mean = 0, SD = 1) soil properties (pH, available phosphorus, carbon, nitrogen, and calcium). All distance metrics were calculated independently for each elevational gradient.

Then, we employed linear mixed‐effects models through the *lme4::lmer* R function (Bates *et al*., 2015) to assess the influence of climate differences on community similarity under homogeneous and heterogeneous vegetation. Log‐transformed fungal community similarity served as the dependent variable, while the continuous variable climatic distance, the categorical variable vegetation type (homogeneous vs heterogeneous), and their interaction were included as fixed explanatory variables. Soil properties distance was also incorporated as a covariate to account for its potential confounding effect. The location of each gradient was included as a random effect to account for potential spatial variation. Because each pairwise similarity and distance value is derived from two observations in the original dataset, pairwise observations are not fully independent and may inflate the effective degrees of freedom. Therefore, *P*‐values were obtained using 100 bootstrap iterations implemented through the sjPlot::tab_model function with bootstrap support (Lüdecke, 2024). We excluded two gradients (Manchoya and Taygetos) from all analyses focused on root endophytes due to the low relative abundances of root endophytes in these two gradients.

A significant interaction between climatic distance and vegetation type, with stronger climatic responses observed under heterogeneous vegetation, was interpreted as evidence that climate‐driven changes in dominant vegetation composition contribute to fungal community turnover (Scenario B in Fig. 1c). Under this scenario, fungal guilds strongly associated with dominant vegetation are expected to exhibit greater compositional turnover along climatic gradients accompanied by vegetation change than along gradients where dominant vegetation remains relatively constant. By contrast, similar responses to climatic distance in both vegetation types (Scenario A in Fig. 1c) would suggest that dominant vegetation turnover contributes less to the observed patterns and that fungal community turnover is primarily associated with climatic variation occurring independently of changes in dominant vegetation composition.

#### OTU‐level analyses

To complement community‐level analyses, we assessed whether species‐level responses to climatic gradients differ between homogeneous and heterogeneous vegetation. For that, the response of each OTU to climate was modeled independently for each altitudinal gradient (eight models in total) using joint species distribution modeling (SDM) through the R package hmsc (Tikhonov *et al*., 2020, 2024), which constructs a Bayesian hierarchical model in the GLM framework (Ovaskainen & Abrego, 2020). For each gradient and Hmsc model, we selected fungal OTUs occurring in at least five samples within the gradient. Climatic variation was represented by the first principal component (PC1_climate), derived from a PCA of MAT_calibrated and MAP. Similarly, soil variation was summarized by the first principal component (PC1_soil), based on a PCA of pH, available phosphorus (P avail.), carbon (C), nitrogen (N), and calcium (Ca). All predictors were scaled to mean 0 and unit variance to have the beta parameters in comparable SD scale. PC1_climate captured over 60% of the variation in climate, while PC1_soil accounted for more than 40% of the variation in soil properties. The response matrix of each Hmsc consisted of the presence‐absence of the OTUs, which were modeled using a binomial model with a probit link function. As fixed explanatory variables, we included the above‐mentioned PC1_climate and PC1_soil, as well as log_10_‐transformed sequencing depth to account for differences in sequencing effort across samples. It should be noted that elevational gradients with homogeneous and heterogeneous vegetation were modeled independently and, therefore, the influence of mediation through dominant vegetation was assessed in a subsequent analysis (see next paragraph). We fitted the models assuming the default priors and sampled the posterior distribution by running four Markov Chain Monte Carlo (MCMC) chains, each of which was run for 3750 iterations, of which 1250 were discarded as burn‐in. We thinned by 10 to obtain a total of 250 posterior samples per chain and 1000 posterior samples in total (Dataset S1). We assessed MCMC convergence using the potential scale reduction factor for the beta parameters, which quantify species responses to environmental covariates (Dataset S2).

Beta parameter values from each Hmsc, quantifying species responses to climate within each gradient, were subsequently extracted for each fungal OTU and converted to absolute values (Dataset S3). We used the absolute values of the coefficients because our hypothesis concerned the strength of species responses to climate, irrespective of whether those responses were positive or negative. Absolute beta values were pooled within each ecological guild. Then, we used linear mixed‐effects models with the vector of absolute beta values as a response variable and vegetation type as a fixed explanatory variable. Raw beta values showed severely right‐skewed distributions and were log_10_‐transformed to ensure normality and homoscedasticity of residuals assumed for the Gaussian mixed‐effects models. Location and OTU identity were included as random effects to account for gradient‐ and OTU‐identity‐specific variations.

The models evaluated the influence of vegetation type (homogeneous vs heterogeneous) on mean absolute beta values, which captured the magnitude of OTU responses to climatic distance. A significantly higher mean absolute beta value in heterogeneous vegetation compared to homogeneous vegetation was interpreted as evidence that climate‐driven changes in dominant vegetation composition contribute to species‐level responses along climatic gradients (Scenario B in Fig. 1c). By contrast, similar mean absolute beta values between vegetation types were interpreted as evidence that dominant vegetation turnover contributes less to the observed species‐level responses (Scenario A in Fig. 1c).

## Results

### Overview of fungal community composition

We obtained a total of 1915 225 fungal sequences, which were clustered into 13 713 OTUs. Of these, 52.4% (representing 69.9% of all sequences) were assigned to one of the focal fungal guilds. Fungal communities were dominated by ECM and soil saprotrophic fungi, followed by litter and wood saprotrophs (Table 1). Plant pathogens and root endophytes were comparatively less abundant.

**Table 1:** Overview of the sequencing dataset's composition based on assigned fungal ecological groups.

Ecological guild	No of OTUs (% OTUs)	No. of sequences (% sequences)	OTUs shared between vegetation types (% sequences)
Ectomycorrhizal fungi	2109 (15.4)	620 523 (32.4)	646 (71.1)
Plant pathogens	540 (3.9)	12 290 (0.6)	236 (77.1)
Root endophytes	220 (1.6)	12 688 (0.7)	93 (27.1)
Soil saprotrophs	2409 (17.6)	512 824 (26.8)	1184 (9.2)
Litter saprotrophs	1088 (7.9)	115 374 (6.0)	467 (15.5)
Wood saprotrophs	814 (5.9)	66 103 (3.4)	332 (17.7)
Others	6533 (47.6)	576 423 (30.1)	na

na, not applicable; OTU, operational taxonomic unit.

The species‐level analyses were based on a subset of OTUs occurring in at least five samples per transect (SDM dataset; Table 1). Across fungal guilds, most OTUs included in the SDM dataset occurred in both homogeneous and heterogeneous vegetation gradients, indicating substantial overlap in species composition between gradient types.

### Community‐level responses

By analyzing the compositional turnover of fungal communities, we found a significant decline in community similarity with climatic distance across all studied fungal ecological guilds (Figs 2, S3; Table S3). However, the extent to which fungal community turnover was mediated by climate‐driven changes in dominant vegetation composition differed among fungal ecological guilds, suggesting distinct responses to dominant vegetation turnover along climatic gradients. Therefore, we categorized the response of fungal ecological guilds into two different groups, corresponding to the predefined A and B scenarios (Fig. 1c).

The first group, corresponding with Scenario A, consisted of ECM fungi only and showed a similar decline in community similarity along climatic distance for both heterogeneous and homogeneous vegetation (Figs 2, S3, Table S3). This pattern suggests that turnover in dominant vegetation within the range of host tree species occurring across the study sites contributes relatively little to ECM fungal community turnover along climatic gradients. Although we found no significant interaction between climatic distance and vegetation type (*P*‐value = 0.4), ECM fungal communities showed consistently higher community dissimilarity along altitudinal transects with heterogeneous than homogeneous vegetation (*P*‐value = 0.02; Figs 2, S3; Table S3).

The second group, corresponding with Scenario B, consisted of plant pathogens, root endophytes, soil saprotrophs, litter saprotrophs, and wood saprotrophs, and showed a significant interaction between climatic distance and vegetation type (Figs 2, S3; Table S3). This pattern indicates that climate‐driven changes in dominant vegetation composition contribute to the turnover of these fungal communities along climatic gradients.

When comparing the strength of community response to climatic distance across fungal ecological guilds, ECM fungi were significantly more responsive to climatic differences than all saprotrophic groups (soil, litter, and wood saprotrophs), as shown in the left panel of Fig. 2b. By contrast, responses of plant pathogens and root endophytes to climate did not differ significantly from either ECM fungi or saprotrophs. Similarly, when comparing the contribution of climate‐driven vegetation turnover among fungal guilds, wood saprotrophs and root endophytes responded more strongly than soil and litter saprotrophs, while plant pathogens again showed no significant difference from either group (right panel of Fig. 2b). Statistical significance was determined based on non‐overlapping 95% confidence intervals (Table S4).

### OTU‐level responses

**Fig. 3:**
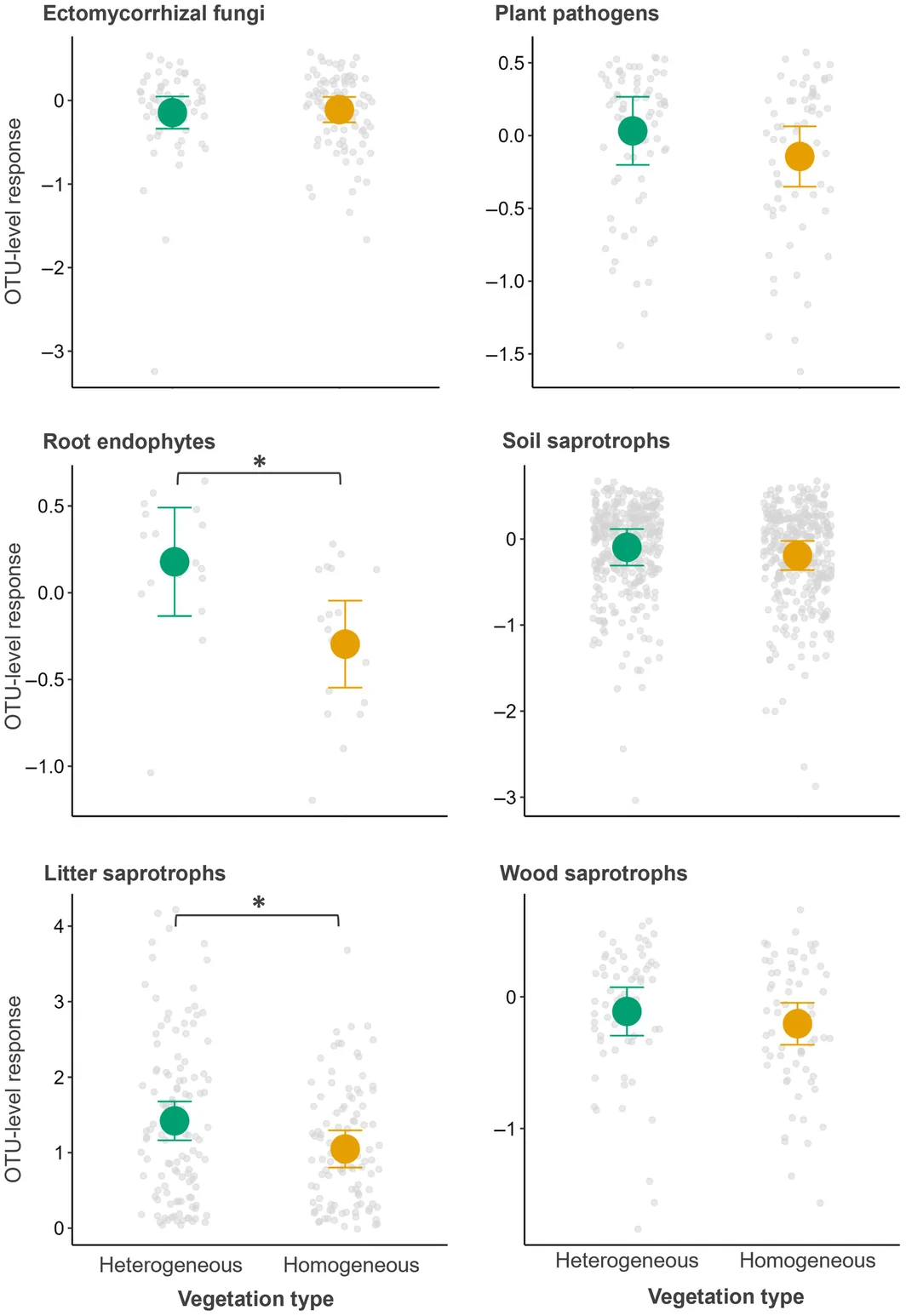
Differences in model‐predicted log_10_‐transformed absolute responses of fungal operational taxonomic units (OTUs) to climate between heterogeneous and homogeneous vegetation across different ecological groups. The figure shows the mean species response ±95% confidence interval, with yellow indicating homogeneous vegetation gradients and green representing heterogeneous vegetation gradients. Asterisks denote significant differences between vegetation types. Points represent observed species‐level responses (absolute beta values) derived from HMSC models, quantifying the magnitude of OTU responses to climate along the altitudinal gradients.

Altogether, we ran eight Hmsc models to identify the distribution of 765 fungal OTUs along the studied altitudinal gradients (model convergence diagnostics are shown in Fig. S4). We then extracted, from every single Hmsc model, beta values quantifying OTU‐level responses to climate. The beta values were converted to absolute values to capture the magnitude of the response, regardless of the direction. The difference in the average magnitude of species’ responses to climate between gradients with homogeneous and heterogeneous vegetation varied between fungal ecological groups (Fig. 3). This suggests that fungal ecological guilds differ in the extent to which species‐level responses along climatic gradients are associated with climate‐driven changes in dominant vegetation composition. The OTU‐level response to climate under homogeneous and heterogeneous vegetation was again categorized into the predefined A and B scenarios (Fig. 1c).

The pattern corresponding to Scenario A was again observed in ECM fungi. No significant difference was observed in OTU responses between homogeneous and heterogeneous vegetation. This indicates that turnover in dominant vegetation contributes relatively little to species‐level responses of ECM fungi along climatic gradients (Fig. 3; Table S5). Similarly, plant pathogens, soil saprotrophs, and wood saprotrophs also exhibited no significant difference in OTU responses between homogeneous and heterogeneous vegetation. Still, they showed a tendency for stronger responses to climate under heterogeneous vegetation compared to ECM fungi (Fig. 3; Table S5).

The second pattern corresponding to Scenario B was observed in root endophytes and litter saprotrophs. In these cases, significantly stronger OTU responses to climate under heterogeneous vegetation were observed. This suggests that climate‐driven changes in dominant vegetation composition contribute substantially to species‐level responses in these groups (Fig. 3; Table S5).

At the OTU level, these patterns largely mirrored broader community‐level trends: ECM fungi showed similar responses under homogeneous and heterogeneous vegetation, whereas root endophytes and litter saprotrophs exhibited stronger responses under heterogeneous vegetation. For the remaining fungal guilds, the contribution of climatic effects mediated by vegetation turnover was less clear, with non‐significant trends suggesting that vegetation turnover may still influence species‐level responses along climatic gradients.

## Discussion

Our study demonstrates that climate and vegetation influence fungal communities in fundamentally different ways across ecological guilds. By comparing elevational gradients with relatively constant dominant vegetation composition (homogeneous vegetation) and gradients characterized by strong dominant vegetation turnover (heterogeneous vegetation), we evaluated the extent to which climate‐mediated changes in dominant vegetation composition contribute to fungal community turnover. We found that ectomycorrhizal (ECM) fungi exhibited similar responses under homogeneous and heterogeneous vegetation, suggesting that turnover in dominant vegetation contributes less to their responses than originally expected. By contrast, plant pathogens, root endophytes, and saprotrophic fungi showed stronger responses under heterogeneous vegetation, indicating a greater association with climate‐mediated vegetation turnover. These contrasting responses reveal fundamental ecological differences among fungal guilds and provide a basis for more realistic predictions of fungal community responses to ongoing climate change.

### Climate‐driven vegetation turnover and fungal guild responses

#### ECM fungi: strong responses to climatic gradients and limited influence of dominant vegetation turnover

We observed pronounced shifts in ECM fungal community composition along climatic gradients, confirming earlier studies that identified temperature and precipitation as major determinants of ECM fungal biogeographic patterns (Miyamoto *et al*., 2018; Větrovský *et al*., 2019; Baldrian *et al*., 2022). By explicitly contrasting gradients with homogeneous and heterogeneous vegetation, our study further demonstrates that ECM fungal communities respond similarly to climatic gradients regardless of whether dominant vegetation composition remains relatively constant or changes with elevation. This pattern was consistent across both community‐ and species‐level analyses, suggesting that turnover in dominant vegetation contributes less to ECM fungal responses than originally expected. Potential limitations regarding the transferability of this finding to ecosystems with different ECM host assemblages are discussed below (see ‘Study limitations and future directions’ section).

Among all examined fungal ecological guilds, ECM fungi showed the strongest response to climatic variation, with significantly steeper community turnover than any saprotrophic group (soil, litter, and wood saprotrophs). This pronounced response is consistent with previous observations that ECM fungi occupy relatively narrow realized climatic niches (Větrovský *et al*., 2019). We initially hypothesized that these patterns would largely reflect the dependence of ECM fungi on climate‐driven changes in vegetation composition. However, the similar responses observed in homogeneous and heterogeneous vegetation gradients suggest that turnover in dominant vegetation contributes less to ECM fungal responses than expected. Consequently, climatic variables appear to provide substantial predictive power for ECM fungal distributions, supporting the use of climate‐based models for forecasting future changes in this guild (Steidinger *et al*., 2020; Tedersoo *et al*., 2022; Baldrian *et al*., 2023). While ECM fungi responded similarly to climatic gradients under homogeneous and heterogeneous vegetation, our data also reveal notable vegetation‐related variability at sites with no climatic differences (climate distance = 0), suggesting that local vegetation heterogeneity may still influence ECM community assembly through stochastic or host‐related processes. Along the gradients with heterogeneous vegetation, tree species composition not only shifted with elevation but also partly varied among sampling plots within the same elevational level. By contrast, along homogeneous vegetation gradients, a single tree species consistently dominated both across the entire gradient and within individual elevation levels (Tables S1 and S2). Contrasting effects of vegetation on different spatial scales likely originate from specific assembly processes of ECM fungal communities. Although ECM fungal communities can certainly be affected by the composition of host plant vegetation (Tedersoo *et al*., 2024), there are only a few ECM fungal species with strict host plant specificity (Põlme *et al*., 2018). Therefore, the effect that vegetation has on ECM fungal communities cannot be easily attributed to the high specificity of mycorrhizal fungi–host plant interactions. Compared to other fungal ecological groups, ECM fungal communities are experiencing higher turnover on a small spatial scale with relatively small species overlaps even between closely located sites (Põlme *et al*., 2013; Martinović *et al*., 2021). Such a high beta diversity of ECM fungal communities is usually attributed to the important role of stochastic processes in their assembly, as previously documented by high dispersal limitations (Peay *et al*., 2010, 2012) and the importance of the priority effect (Kennedy *et al*., 2009). High vegetation variability on the local scale can enhance the role of assembly processes such as the priority effect. This is because differences in host plant phenology lead to asynchronous root growth patterns, which in turn result in distinct ECM fungal communities at small spatial scales in heterogeneous ECM vegetation. Although these processes can amplify the effect of vegetation on the higher stochasticity of ECM fungal communities on a small scale, they would have little effect on large spatial scales. Thus, stochastic factors could contribute to the observed variability in ECM fungal communities across plots with similar climatic and vegetational characteristics, without substantially altering the broader responses of ECM fungal communities to climatic gradients.

#### Plant pathogens and root endophytes: strong association with climate‐driven vegetation turnover

In contrast to ECM fungi, communities of plant pathogens were strongly associated with effects mediated by dominant vegetation turnover. Climatic factors such as temperature and humidity are known to drive pathogen outbreaks and distributions (Delgado‐Baquerizo *et al*., 2020; Raza & Bebber, 2022; Romero *et al*., 2022), but our results suggest that changes in dominant vegetation composition contribute substantially to pathogen community turnover along climatic gradients. The replacement of drought‐sensitive plants by more tolerant species restructures pathogen communities due to the high host specificity of many plant–pathogen interactions (Delavaux *et al*., 2021; Li *et al*., 2023; Kumar & Mukhopadhyay, 2025). Consequently, predictive models relying solely on climatic variables may substantially underestimate pathogen community shifts under future climates.

Root endophytes showed a similar pattern, with stronger responses under heterogeneous vegetation than under homogeneous vegetation. Despite their generally low host specificity (Jumpponen & Trappe, 1998; Põlme *et al*., 2018), their responses to vegetation change were even stronger than those of soil and litter saprotrophs, highlighting their dependence on host plant composition. These findings underline the key role of vegetation in shaping root endophytic communities and the need to incorporate vegetation dynamics into predictive models of their climate responses (Brigham *et al*., 2023; Wang *et al*., 2023; Edwards *et al*., 2025).

#### Saprotrophic fungi: association with climate‐driven vegetation turnover and substrate availability

Contrary to our initial hypothesis, saprotrophic fungal communities showed stronger responses under heterogeneous vegetation across all examined subgroups (soil, litter, and wood saprotrophs). These effects likely arise from their dependence on vegetation‐controlled environmental conditions, including variation in litter chemistry, substrate availability, and nutrient composition (Urbanová *et al*., 2015; Barbi *et al*., 2016; Baldrian *et al*., 2023). Such vegetation‐mediated responses to climate are consistent with experimental evidence showing interactive effects of vegetation composition and climate on decomposer communities (Dawson‐Glass *et al*., 2023).

The strength of the association with vegetation turnover differed among saprotrophic guilds. Wood saprotrophs responded most strongly, likely reflecting their dependence on host tree identity and wood properties such as pH, moisture, and nutrient content, which vary among species and shape fungal community composition (Lepinay *et al*., 2022). Litter saprotrophs also showed a pronounced response, supported consistently at both community and species levels, in agreement with studies demonstrating strong effects of litter identity and diversity on fungal richness and turnover (Otsing *et al*., 2018). By contrast, soil saprotrophs exhibited the weakest mediation by dominant vegetation turnover. Given the central role of saprotrophic fungi in decomposition and nutrient cycling, even moderate shifts in these groups, particularly in litter and wood decomposers, could substantially affect ecosystem functioning under future climate scenarios (Pec *et al*., 2021). Together, these results highlight the importance of incorporating vegetation dynamics alongside climatic variables into predictive frameworks to better capture saprotrophic fungal dynamics under global change.

### Study limitations and future directions

Although our study provided robust evidence for distinct climatic responses among fungal guilds, several limitations must be acknowledged when interpreting our results. This study focused exclusively on ECM‐dominated forests in Europe, characterized by relatively low variation in plant community composition and a limited number of dominant host species. Although ECM fungi generally exhibit low host plant specificity, our findings may have limited transferability to forests with different ECM hosts (Tedersoo *et al*., 2014).

An additional source of indirect climatic influence that was not explicitly quantified in this study is understory vegetation. Understory plant communities may represent an important pathway through which climate indirectly affects fungal communities, as they often respond rapidly to local climatic, edaphic, and microtopographic variation. Changes in understory composition may modify litter inputs, root exudation patterns, and microclimatic conditions at the forest floor, thereby influencing soil fungal community structure. Although these mechanisms were beyond the scope of the present study and were not directly assessed here, recent work has shown that variation in understory vegetation composition and diversity can substantially affect soil fungal communities (Odriozola *et al*., 2021).

Beyond vegetation‐mediated pathways, other indirect effects of climate may operate through changes in soil physicochemical properties. In particular, soil texture – which strongly influences soil moisture availability and microclimatic stability (Xia *et al*., 2020; Patel *et al*., 2021)—was not measured in this study, despite its potential to shape both fungal community composition and the strength of climate–vegetation–soil interactions. Heterogeneity in unmeasured soil properties, including texture and associated moisture dynamics, may therefore contribute to additional unexplained variability in the observed patterns and should be addressed in future research.

Part of the variation attributed to climatic effects in our models may also reflect changes in forest productivity that co‐vary with altitude. Forest productivity often declines with increasing elevation due to lower temperatures and shorter growing seasons (Rana *et al*., 1989; Hernández Gordillo *et al*., 2021), although this relationship can vary among ecosystems and climatic zones, with some studies reporting stable or even higher productivity at mid‐ or high‐elevation sites where water availability is greater or drought stress is reduced (Wei *et al*., 2018; Knowles *et al*., 2020). As forest productivity was not directly measured in this study, its potential contribution to the observed climate‐related patterns cannot be fully disentangled. Moreover, forest productivity is closely linked to belowground carbon allocation, influencing both symbiotic and saprotrophic fungi through changes in root‐derived carbon inputs such as exudates and litter production (Litton *et al*., 2007; Phillips *et al*., 2013).

Future studies should aim to disentangle these multiple indirect pathways by integrating detailed measurements of vegetation structure (including understory communities), soil properties, and ecosystem productivity across climatic gradients. Integrating both community‐ and species‐level perspectives across a wider range of ecosystems, vegetation types, and fungal associations will be essential for improving predictions of fungal responses to climate change. In particular, we recommend the development of predictive models that combine climatic niche information with vegetation characteristics and soil parameters, especially for fungal groups whose responses to climate are strongly mediated by vegetation turnover. Such integrative frameworks are essential to capture the complexity of fungal ecology and provide more accurate forecasts of biodiversity and ecosystem function under global change.

### Conclusions

Our results reveal fundamental differences among fungal ecological guilds in how their communities respond to climatic gradients and climate‐driven vegetation turnover. Ectomycorrhizal (ECM) fungi showed strong compositional turnover along climatic gradients regardless of whether dominant vegetation composition remained relatively constant or changed with elevation, suggesting that turnover in dominant vegetation contributes less to their responses than originally expected. By contrast, plant pathogens, root endophytes, and saprotrophic fungi exhibited stronger responses under heterogeneous vegetation, indicating a greater influence of the effects mediated by vegetation turnover on their community composition.

These contrasting patterns demonstrate that fungal guilds differ substantially in the extent to which their distributions are associated with vegetation turnover along climatic gradients. Consequently, predictive models based solely on climatic variables may provide useful forecasts for some fungal guilds, particularly ECM fungi, whereas incorporating vegetation dynamics is likely to improve predictions for guilds whose responses are more strongly associated with climate‐driven changes in vegetation composition.

## Competing interests

None declared.

## Author contributions

PK coordinated this project. A Moravcová and PK designed the study. A Moravcová, IO, TM, VB, LM, ET and PK participated in the fieldwork. A Moravcová, TM and PK did data curation. A Moravcová and IO analyzed the data. A Moravcová wrote the original draft. FB, TM, IO, A Machač, PB and PK reviewed the first draft. All the authors reviewed and contributed to the final version of the manuscript.

## Disclaimer

The New Phytologist Foundation remains neutral with regard to jurisdictional claims in maps and in any institutional affiliations.

## Supporting information


**Dataset S1** R script for defining and fitting the gradient‐specific Hmsc models; the file can be opened and run using R or RStudio.
**Dataset S2** R script for assessing MCMC convergence of the fitted Hmsc models; the file can be opened and run using R or RStudio.
**Dataset S3** R script for extracting OTU‐level beta parameter estimates from the fitted Hmsc models; the file can be opened and run using R or RStudio.

**Fig. S1** Environmental characteristics of the studied elevational gradients.
**Fig. S2** Comparison of mean annual temperature estimates from climate databases and *in situ* measurements.
**Fig. S3** Relationship between climatic distance and fungal community similarity across fungal ecological guilds.
**Fig. S4** Convergence diagnostics of HMSC models.

**Table S1** Sampling site information.
**Table S2** Tree canopy composition of sampling plots.
**Table S3** Results of community‐level mixed‐effects models.
**Table S4** Bootstrap confidence intervals for community‐level analyses.
**Table S5** Results of OTU‐level mixed‐effects models.
Please note: Wiley is not responsible for the content or functionality of any Supporting Information supplied by the authors. Any queries (other than missing material) should be directed to the *New Phytologist* Central Office.

## Data Availability

Full sequencing data are deposited in the NCBI Sequence Read Archive (SRA) under BioProject PRJNA869039. The R scripts used to define and fit the Hmsc models, assess MCMC convergence, and extract beta parameter estimates are provided in Datasets S1–S3. All other data supporting the findings of this study are included in this published article and its Supporting Information files.
