# Supplementary material for: Differential responses of fungal guilds to climatic effects mediated by changes in dominant vegetation
Source: New Phytol. 2026 Aug 24;252(2):861–74. doi: 10.1111/nph.71523 (PMC13583400; doi:10.1111/nph.71523)
Supplement: Supplementary file 1 [file NPH-252-861-s001.docx]

## *New Phytologist* Supporting Information

Article title: Differential responses of fungal guilds to climatic effects mediated by changes in dominant vegetation

Authors: Andrea Moravcová, Florian Barbi, Iñaki Odriozola, Tijana Martinović, Vendula Brabcová, Lenka Mészárošová, Ella Thoen, Antonin Machac, Petr Baldrian, Petr Kohout

Article acceptance date: 2 August 2026

The following Supporting Information is available for this article:

**Fig. S1** Environmental characteristics of the studied elevational gradients.

**Fig. S2** Comparison of mean annual temperature estimates from climate databases and in situ measurements.

**Fig S3.** Relationship between climatic distance and fungal community similarity across fungal ecological guilds.

**Fig. S4** Convergence diagnostics of HMSC models.

**Table S1** Sampling site information (Excel file submitted separately).

**Table S2** Tree canopy composition of sampling plots.

**Table S3** Results of community-level mixed-effects models.

**Table S4** Bootstrap confidence intervals for community-level analyses.

**Table S5** Results of OTU-level mixed-effects models.

**Dataset S1** R script for defining and fitting the gradient-specific Hmsc models; the file can be opened and run using R or RStudio.

**Dataset S2** R script for assessing MCMC convergence of the fitted Hmsc models; the file can be opened and run using R or RStudio.

**Dataset S3** R script for extracting OTU-level beta parameter estimates from the fitted Hmsc models; the file can be opened and run using R or RStudio.

**Fig. S1** Environmental characteristics of the studied elevational gradients. Minimum, mean, and maximum values of environmental and vegetation variables across the eight studied gradients. Circles, triangles, and squares represent minimum, mean, and maximum values, respectively. Solid lines correspond to gradients with heterogeneous vegetation, and dashed lines to gradients with homogeneous vegetation. Colors indicate locations: Taygetos (black), Manchoya (yellow), Cvrsnica (turquoise), Comborsier (green), Krn (blue), Dachstein (orange), Malá Fatra (pink), and Jondal (purple). Variables include altitude, mean annual temperature (MAT), mean annual precipitation (MAP), soil pH, total carbon, nitrogen, available phosphorus, and calcium.

**Fig. S2** Comparison of mean annual temperature (MAT) estimates from different sources across three mountain sites: (A) Dachstein (Austria), (B) Comborsier (France), and (C) Krn (Slovenia). The data include MAT estimates from climate databases (CHELSA_MAT, ForestClim_MAT) and in situ measurements using TMS probes at three heights: 15 cm above the ground (TMS_air15_MAT), 2 cm above the ground (TMS_air2_MAT), and 2 cm in the soil (TMS_soil_MAT). Additionally, CHELSA_MAT_calibrated represents the CHELSA-derived MAT values adjusted for altitude to better align with the trend observed from TMS measurements. The in situ TMS data were recorded over different periods: Dachstein (09/25/2022–09/25/2023), Krn (06/05/2021–06/05/2022), and Comborsier (07/14/2022–07/14/2023).

**Fig. S3** Relationship between climatic distance and fungal community similarity along altitudinal gradients in heterogeneous (green) and homogeneous (yellow) vegetation, shown separately for each fungal ecological group. Lines represent model-predicted relationships, with shaded areas indicating 95% confidence intervals. Points correspond to observed pairwise similarities between plots and thus represent real data.

**Fig. S4** Convergence diagnostics of HMSC models. Potential scale reduction factors (PSRF) for beta parameters across all eight studied gradients (Alps, Cvrsnica, Dachstein, Fatra, Jondal, Krn, Pyrenees, Taygetos). All PSRF values were close to 1, indicating good convergence of the four Markov Chain Monte Carlo (MCMC) chains used to estimate species responses to environmental covariates.

**Dataset S1**. Definition and fitting of the Hmsc models. R script used to import the fungal OTU and environmental data and define and fit eight gradient-specific Bayesian joint species distribution models using Hmsc. The models include PC1_climate, PC1_soil, and log-transformed sequencing depth as fixed explanatory variables and Plot as a random effect. The file can be opened and run in R or RStudio and requires the R packages Hmsc and readxl.

**Dataset S2**. Evaluation of Hmsc model convergence. R script used to calculate the potential scale reduction factor for the beta parameters of the fitted Hmsc models and to produce the MCMC convergence diagnostic plot. The file can be opened and run in R or RStudio and requires the R packages Hmsc, coda, readxl, and vioplot.

**Dataset S3**. Extraction of Hmsc parameter estimates. R script used to extract posterior mean beta coefficients describing OTU-level responses to PC1_climate from the fitted Hmsc models and export the resulting estimates as a CSV file. The file can be opened and run in R or RStudio and requires the R packages Hmsc and readxl.
